# Blood-Based miRNA Biomarkers as Correlates of Brain-Based miRNA Expression

**DOI:** 10.3389/fnmol.2022.817290

**Published:** 2022-03-22

**Authors:** Mark Z. Kos, Sobha Puppala, Dianne Cruz, Jennifer L. Neary, Ashish Kumar, Emma Dalan, Cun Li, Peter Nathanielsz, Melanie A. Carless

**Affiliations:** ^1^Department of Human Genetics, South Texas Diabetes and Obesity Institute, University of Texas Rio Grande Valley School of Medicine, Edinburg, TX, United States; ^2^Department of Internal Medicine-Section of Molecular Medicine, Wake Forest Baptist Medical Center, Winston-Salem, NC, United States; ^3^Duke University School of Medicine, Durham, NC, United States; ^4^Department of Computational Biology, St. Jude Children’s Research Hospital, Memphis, TN, United States; ^5^Department of Biology, University of Texas at San Antonio, San Antonio, TX, United States; ^6^Southwest National Primate Research Center, Texas Biomedical Research Institute, San Antonio, TX, United States; ^7^Department of Animal Science, University of Wyoming, Laramie, WY, United States; ^8^Population Health, Texas Biomedical Research Institute, San Antonio, TX, United States

**Keywords:** baboon, blood, brain, correlation, miRNA expression, sequencing

## Abstract

The use of easily accessible peripheral samples, such as blood or saliva, to investigate neurological and neuropsychiatric disorders is well-established in genetic and epigenetic research, but the pathological implications of such biomarkers are not easily discerned. To better understand the relationship between peripheral blood- and brain-based epigenetic activity, we conducted a pilot study on captive baboons (*Papio hamadryas*) to investigate correlations between miRNA expression in peripheral blood mononuclear cells (PBMCs) and 14 different cortical and subcortical brain regions, represented by two study groups comprised of 4 and 6 animals. Using next-generation sequencing, we identified 362 miRNAs expressed at ≥ 10 read counts in 80% or more of the brain samples analyzed. Nominally significant pairwise correlations (one-sided *P* < 0.05) between peripheral blood and mean brain expression levels of individual miRNAs were observed for 39 and 44 miRNAs in each group. When miRNA expression levels were averaged for tissue type across animals within the groups, Spearman’s rank correlations between PBMCs and the brain regions are all highly significant (*r_*s*_* = 0.47–0.57; *P* < 2.2 × 10^–16^), although pairwise correlations among the brain regions are markedly stronger (*r_*s*_* = 0.86–0.99). Principal component analysis revealed differentiation in miRNA expression between peripheral blood and the brain regions for the first component (accounting for ∼75% of variance). Linear mixed effects modeling attributed most of the variance in expression to differences between miRNAs (>70%), with non-significant 7.5% and 13.1% assigned to differences between blood and brain-based samples in the two study groups. Hierarchical UPGMA clustering revealed a major co-expression branch in both study groups, comprised of miRNAs globally upregulated in blood relative to the brain samples, exhibiting an enrichment of miRNAs expressed in immune cells (CD14+, CD15+, CD19+, CD3+, and CD56 + leukocytes) among the top blood-brain correlates, with the gene *MYC*, encoding a master transcription factor that regulates angiogenesis and neural stem cell activation, representing the most prevalent miRNA target. Although some differentiation was observed between tissue types, these preliminary findings reveal wider correlated patterns between blood- and brain-expressed miRNAs, suggesting the potential utility of blood-based miRNA profiling for investigating by proxy certain miRNA activity in the brain, with implications for neuroinflammatory and c-Myc-mediated processes.

## Introduction

MicroRNAs (miRNAs) are a group of endogenous, evolutionarily conserved small non-coding RNAs that post-transcriptionally regulate gene expression through mRNA destabilization and translational repression (Eichhorn et al., 2014; Santpere et al., 2016). They play an important role in human brain development and in the pathogenesis of neurodevelopmental, neurodegenerative, and neuropsychiatric disorders [reviewed in Barry (2014)]. Further, almost 50% of miRNAs are expressed in the brain, and their putative target genes are involved in the regulation of basic neural processes, such as neurogenesis and neuroplasticity (Ziats and Rennert, 2014).

Much of the direct evidence implicating miRNAs in neurological processes is derived from knockout studies in rodents (Davis et al., 2008; Stark et al., 2008), which can be advantageous in characterizing causal effects. However, rodent models of brain-related disease often do not accurately reflect human disease symptomatology [reviewed in Nestler and Hyman (2010)] or the complexity of the human brain (Defelipe, 2011), exhibiting altered patterns of evolutionary conservation for genes underlying human psychiatric disorders (Ogawa and Vallender, 2014) and a high rate of divergence (from humans) in brain miRNA expression (Roux et al., 2012). Human studies, on the other hand, allow for a more direct investigation of human neurology through analysis of miRNA expression in peripheral tissues (e.g., blood, saliva) of living individuals, post-mortem analysis of miRNA expression in the brain, or functional analysis in cell-based neuronal models of disease. But each of these study designs have limitations. Population-based studies can be used to assess peripheral miRNA biomarkers of disease in large, and potentially drug-naïve, cohorts and associate these with measurable phenotypes or symptoms of disease; however, peripheral biomarkers may not be representative of the tissue of interest (Cai et al., 2010) and causality cannot be easily determined. Analyses of post-mortem tissue are challenging and complicated by their typically small sample sizes and factors such as manner of death, postmortem interval, tissue pH, and RNA integrity (McCullumsmith and Meador-Woodruff, 2011). And more importantly, they are not designed to differentiate causal miRNA expression signatures from those that result from progression of the disease or from death. And lastly, the recent advent of methods to derive neuronal cells and brain organoids from pluripotent stem cells [reviewed in Adegbola et al. (2017) and McKinney (2017)] allows the investigation of patient samples that are representative of brain tissue; but development and characterization of these models are cost-prohibitive on a large scale and their relation to brain tissue sources, especially with respect to miRNA expression networks, is not yet well defined.

A combination of these approaches, each with their own strengths, will be essential in determining the complex underlying biology of neurological and neuropsychiatric disorders, particularly with respect to transcriptomic and epigenomic architectures. To bridge the gap between studies of peripheral miRNA expression (from blood) and post-mortem analysis of brain miRNA expression, we conducted a pilot study to examine correlations in matched samples from blood and 14 different brain regions in two groups of baboons (*n* = 4 and *n* = 6), a primate species with genomic, physiological, and neuroanatomical similarities to humans that serves as a well-suited model for studying the genetics and epigenetics of complex human phenotypes (Cox et al., 2013). We observed significant correlations between blood and the examined brain regions for tissue-wide patterns in miRNA expression. Co-expression between miRNAs among the different tissue types revealed major clusters of up- and downregulated miRNAs in the blood-based samples, with enrichments of miRNAs expressed in immune cell types among the top blood-brain correlates, as well as miRNA targets functionally annotated to different aspects of gene expression regulation.

## Materials and Methods

### Animal Care and Tissue Collection

All animal procedures were approved by the Texas Biomedical Research Institute (Texas Biomed) Institutional Animal Care and Use Committee (IACUC) and conducted in AAALAC International approved facilities. Brain and blood samples were collected from two groups of baboons (*Papio hamadryas*) (Group A, *n* = 4; Group B, *n* = 6), previously housed at the Southwest National Primate Research Center (SNPRC) located on the Texas Biomed campus. For both groups, animals were enrolled in several research studies throughout their life, which included those related to pregnancy, epilepsy, ophthalmoscopic examination, steroid hormone variation, immunogenicity studies, renal blood flow and derivation of stem cells. Study enrollment and related procedures were different for each animal but were completed well before euthanasia of the animals (except for Group B animals, see below). We do not expect that these studies would have a significant impact on the miRNA transcriptional profiles obtained in this study. As necessitated, animals were admitted to and treated at the SNPRC clinic for injury and illness (primarily treatment of lacerations and monitoring of weight loss) throughout their life and underwent regular routine health screens. Animals in Group A were all female, with a mean age of 13.5 years (range 12.8–14.7 years) and mean weight of 20.3 kg (range 18.5–23.2 kg). Samples were ascertained following routine euthanasia for population management at the SNPRC colony. Animals in Group B included 5 males with a mean age of 18.5 years (range 16.6–22.7 years) and a mean weight of 29.2 kg (range 22.0–35.9 kg), and 1 female, 7.9 years old and weighing 15.0 kg. Group B animals were enrolled in a pregnancy study (as controls) and transported to the University of Texas Health Science Center at San Antonio prior to euthanasia; enrollment in this study is not expected to significantly impact the miRNA profiles generated. The brain regions that had been collected for Group A animals were not available for Group B animals enrolled in this study, and therefore a different set of brain regions were examined between the two study groups.

**FIGURE 1:**
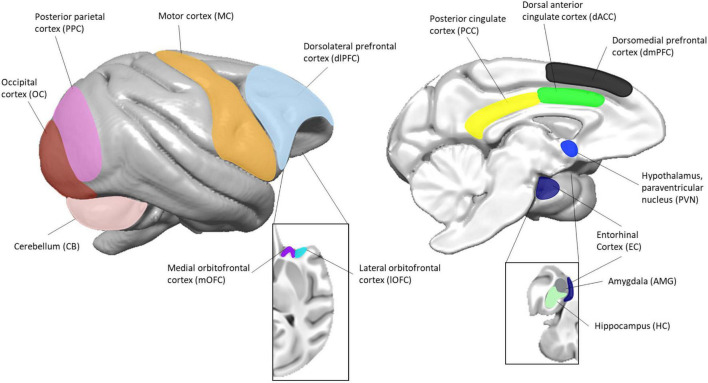
Location of brain regions used in this study. The image to the left represents the lateral view and the image to the right represents the medial view. Insets are of the orbitofrontal (left) and mesial temporal (right) structures. Surface renderings of a published baboon brain template (Love et al., 2016) were created using MANGO (https://ric.uthscsa.edu/mango/).

Baboons were sedated with ketamine (10 mg/kg) prior to collection of blood in BD Vacutainer^®^ CPT™ Mononuclear Cell Preparation Tubes (Sodium Citrate; Becton-Dickinson, Franklin Lakes, NJ, United States), anesthetized (1–2% isoflurane), and euthanized *via* either intravenous injection of pentobarbital (>100 mg/kg; Group A) or exsanguination while under general anesthesia (Group B), in accordance with the guidelines of the American Veterinary Medical Association (AVMA) Panel on Euthanasia [AVMA Panel on Euthanasia, 2001]. Necropsy was performed to obtain brain tissue. We isolated seven different brain regions from each group of animals, as outlined in Table 1 and Figure 1. The brain regions collected from each group of animals are independent. To account for differences between the two study groups (method of euthanasia, brain regions collected), all analyses were done independently for each group.

**TABLE 1:** Description of brain regions examined.

Region	Description
**Group A (*n* = 4)**	
Amygdala (AMG)	Medial temporal lobe, ∼0.5 cm posterior to temporal pole
Hypothalamus (PVN)	Hypothalamus, paraventricular nucleus, ∼1.0 cm posterior to temporal pole, 1 mm dorsal to the optic chiasm, ∼1 mm bilateral bank of third ventricle
Dorsomedial prefrontal cortex (dmPFC)	Dorsomedial bank of the superior frontal gyrus, 1.0 cm posterior to frontal pole
Dorsal anterior cingulate cortex (dACC)	Immediately dorsal to the genu of the corpus callosum, ∼1.5 cm posterior to frontal pole
Lateral orbitofrontal cortex (lOFC)	Orbitofrontal cortex, ∼0.5 cm anterior to the genu of the corpus callosum, ventrolateral frontal cortex
Medial orbitofrontal cortex (mOFC)	Orbitofrontal cortex, ∼3 mm anterior to the genu of the corpus callosum, ventromedial frontal cortex, straight gyrus
Posterior cingulate cortex (PCC)	Immediately dorsal to the splenium of the corpus callosum, ∼2.0 cm anterior to the occipital pole
**Group B (*n* = 6)**	
Hippocampus (HC)	Hippocampus containing all substructures
Cerebellum (CB)	Lateral cerebellar lobule
Dorsolateral prefrontal cortex (dlPFC)	Dorsal bank of the principal sulcus, 1.0–1.5 cm posterior to the frontal pole
Motor cortex (MC)	Within 5 mm anterior to the central sulcus
Entorhinal cortex (EC)	At the level of the thalamic lateral geniculate nucleus
Posterior parietal cortex (PPC)	Dorsal bank of the parietal sulcus (nearest dorsal aspect of brain)
Occipital cortex (OC)	Calcarine sulcus (primary visual cortex), 1 cm anterior to the occipital pole

Peripheral blood mononuclear cells (PBMCs) were isolated from each animal within 2 h of blood collection, according to the manufacturers’ protocol for the BD Vacutainer^®^ CPT™ Mononuclear Cell Preparation Tubes, stored in freezing media (RPMI complete media, 10% FBS, 10% DMSO), and cooled at 1°C per minute in a −80°C freezer before being transferred to liquid nitrogen. Brain regions were dissected according to established brain landmarks and guided by atlases describing the anatomy of human, baboon and rhesus macaque brains (Saleem and Logothetis, 2012; Bakker et al., 2015), as well as knowledge and experience gained over the years. Only the regions of interest were dissected, without contamination from surrounding regions. All samples for both groups were frozen within 2 h of brain collection and therefore have a similar post-mortem interval. For animals in Group A, the whole brain was obtained and kept at 4°C until the time of dissection. The brain was hemisected at the midline into right and left hemispheres. The cerebellum was separated from the cerebrum at the level of the midbrain. The hemispheres were cut in the coronal plane, from anterior to posterior, approximately 0.5 cm in thickness per block. The cerebellum was cut sagittally, approximately 0.5 cm in thickness per block. The subregions (Table 1 and Figure 1) were dissected from each coronal block prior to freezing. Each subregion was placed in a labeled tube, flash-frozen in 2-methylbutane chilled to −60°C and stored at −80°C until experimental use. For animals in Group B, the brain was hemisected and the subregions examined in this study (Table 1 and Figure 1) were dissected directly from fresh tissue based on known anatomical structures and landmarks. They were then placed in a cryovial and flash frozen in liquid nitrogen. All samples were stored in a −80°C freezer prior to RNA extraction. For one animal in Group A (animal A4), we were not able to obtain the amygdala, and thus only three animal samples from this brain region were available.

### RNA Extraction

Total RNA was isolated from peripheral blood cells and the brain regions described in Table 1 using the miRNeasy Mini Kit (Qiagen, Germantown, MD, United States), according to manufacturers’ instructions. Briefly, PBMCs were thawed in a 37°C waterbath and homogenized in QIAzol lysis reagent using a Fisher Scientific PowerGen 125 Handheld Homogenizer (∼10 s homogenization; Fisher Scientific, Waltham, MA, United States). Frozen brain tissue was homogenized in QIAzol lysis reagent using a Branson homogenizer (∼30 s homogenization) until no tissue was visible and then passed through a QIAshredder (Qiagen). Following addition of chloroform, vortexing, and centrifugation, the RNA-containing aqueous phase was separated. Total RNA was precipitated by addition of ethanol, bound to a spin column, and purified through a series of wash buffers. Total RNA was eluted in RNase/DNase free water and integrity assessed *via* the Bioanalyzer 2100 (Agilent Technologies, Santa Clara, CA, United States). The RNA Integrity Number (RIN) ranged from 6 to 10 (85% of samples were RIN ≥ 7), for all but two samples (RINs 4.7 and 5.9). Since miRNAs are extremely stable, a low RIN is not considered indicative of miRNA quality and has been shown to have little to no impact on miRNA expression (Jung et al., 2010; Hall et al., 2012; Sanchez et al., 2018). We therefore included all samples in this study.

### miRNA Sequencing

Small RNA sequencing libraries were prepared using the Illumina Truseq Small RNA Sample Prep Kit. Each library (sample) included a unique index and libraries were pooled after cDNA synthesis. cDNA libraries underwent cluster generation on Illumina’s Cluster Station and were sequenced using the Illumina GAIIx sequencer. Raw sequence reads were obtained using Illumina’s Pipeline v1.5 software following sequencing image analysis by Pipeline Firecrest Module and base calling by Pipeline Bustard Module. The extracted sequence reads were normalized, annotated and abundance determined using mirDeep2 (Friedlander et al., 2012).

Adapter sequences were trimmed and resulting reads were aligned to the human miRNA transcriptome (miRbase v21) (Griffiths-Jones, 2006) with the bowtie aligner in the mirDeep2 package, due to limited miRNA sequences available for the baboon genus; a prior study showed that 95% of baboon miRNA transcripts are identical in sequence to human miRNA transcripts in the liver (Karere et al., 2012). Alignment was stringent, with zero mismatches allowed, thus limiting the identification of novel or species-specific variants. Samples with < 500,000 read counts aligning to miRBase v21 were excluded from the analysis; these were the cerebellum from animal B2, motor cortex from animal B4, and occipital cortex from animal B6. In total, 76 samples were included in our analysis. Since our primary interest is in the utility of blood samples as a biomarker for brain-related pathology, for all analyses, we selected only miRNAs that were expressed (≥10 read counts) in 80% or more of the brain samples analyzed (*n* = 362; Supplementary Table 1). These miRNA expression data have been uploaded to the Gene Expression Omnibus (GEO) repository (accession number GSE190675).

### Statistical Analysis

Pairwise correlation analyses were performed using the R function “cor.test,” with statistical significance set to one-sided *P* < 0.05 (i.e., investigating the *positive* linear relationship in miRNA expression levels between blood and brain samples). Two different sets of pairwise correlations were calculated. First, mean expression levels per miRNA for each tissue type (i.e., PBMC, brain regions) was examined using Spearman’s rank-order approach (due to non-normality in expression data across miRNAs, assessed by the Shapiro–Wilk test), yielding an *overall* correlation between each pair of tissue types. Second, correlations between PBMC expression levels vs. regional and overall mean brain expression levels of each study baboon for *each of the 362 miRNAs* was calculated using the Pearson method. Bonferroni corrections, when applied, are based on an α value of 0.05 divided by the number of tests performed. Principal component analysis (PCA) was performed on normalized miRNA counts from mirDeep2 (per million mapped miRNA reads) using the Partek data analysis software. Three-dimensional (3D) plots of the first three principal components were generated using the R package “rgl” v. 0.100.19.

Variance components analysis was performed on the miRNA expression data by fitting a linear mixed effects model (LMM) using the restricted maximum likelihood (REML) approach. This was performed with the R package “VCA” v. 1.3.4 using the function “remlMM,” which relies on the “lmer” function from “lme4” v. 1.1.21 (Bates et al., 2015). Confidence intervals for variance components were determined using the Satterthwaite approach (Satterthwaite, 1946). Due to the non-normality and variance heterogeneity in the miRNA expression data, logarithmic transformation of the data was performed prior to analysis (Bartlett and Kendall, 1946). Random effects in the LMM were study animal, miRNA, tissue type (i.e., blood vs. brain), and brain region (nested within the tissue type component). Fixed effects were age and sex.

Two-way hierarchical clustering *via* UPGMA (unweighted pair group method with arithmetic mean) and heatmaps of Z-scores for expression of each miRNA were generated in the R package “gplots” v. 3.5.3, with the two dendrograms clustering miRNA expression levels by tissue sample (dissimilarity distance based on Spearman’s correlation coefficient: 1 − *r*_*s*_), representing heatmap columns, and by miRNA (dissimilarity distance based on Pearson correlation coefficient: 1 − *r*), representing heatmap rows.

MiRNA Set Enrichment Analysis (MSEA) was performed on miRNA lists of interest using the web-based application miEAA (Backes et al., 2016), accessible at: http://www.ccb.uni-saarland.de/mieaa_tool/. MSEA is akin to Gene Set Enrichment Analysis (GSEA), in which a running sum statistic is calculated top-to-bottom for lists of miRNAs ordered by some criterion (here correlation coefficients between PBMC miRNA expression and mean brain expression levels); and provides a Kolmogorov–Smirnov-like statistic that quantifies the over-representation of miRNAs in a predefined set at the top or bottom of the tested list. Statistical significance is determined by exact *P*-values adjusted by the Benjamini–Hochberg approach. MSEA tests more than 14,000 categories generated from different miRNA-specific tools and databases (e.g., miRBase, HMDD2, miRWalk) and publications. Additionally, gene targets for miRNAs comprising major UPGMA dendrogram clusters were identified from miRTarBase, a curated database of miRNA-target interactions (Hsu et al., 2011). These were examined for enrichment using the over-representation analysis option in miEAA, using a Fisher’s exact test, with all annotated miRNAs serving as the reference set. Top enriched gene targets (adjusted *P* < 0.01) were then examined with the pathway-mining and functional annotation tool DAVID (Huang et al., 2009).

## Results

In total, we analyzed ∼239 million sequence reads from 76 blood and brain samples, achieving a mapping percentage of 66.3% for the miRbase v21 reference. Four brain samples were excluded due to low mapped read counts (<500,000; *n* = 3) or unavailable tissue (*n* = 1). We identified 362 miRNAs that were expressed (≥10 read counts) in at least 80% of the brain samples analyzed (Supplementary Table 1). We computed Pearson correlations for expression levels of each of the 362 miRNAs between PBMC and the mean of the normalized read counts of the various brain samples in each animal from the two study groups (Group A, *n* = 4; Group B, *n* = 6; Supplementary Table 1). For Group A, the median correlation coefficient (*r*) is 0.27, with 39 miRNAs (11.3%) showing nominally significant correlation (one-sided *P* < 0.05) between blood and brain expression levels. The strongest correlation was observed for hsa-miR-99b-5p (*r* = 1.00; *P* = 2.4 × 10^–5^), the lone significant result after Bonferroni correction for either group (adjusted *P* = 8.4 × 10^–3^). For Group B, median *r* is 0.18, with 44 miRNAs (12.9%) exhibiting nominally significant correlation between blood and brain expression levels, with the top result observed for hsa-miR-194-5p (*r* = 0.97; *P* = 4.9 × 10^–4^; adjusted *P* = 0.17). Only five miRNAs showed nominally significant correlations (*P* < 0.05) between blood and brain expression levels in both groups (hsa-let-7d-5p, hsa-miR-30c-5p, hsa-miR-182-5p, hsa-miR-194-5p and hsa-miR409-5p; Table 2), likely impacted by the small sample sizes and reduced statistical power (Fisher’s Exact test *P* = 0.47).

**TABLE 2:** miRNAs that are nominally correlated between blood and mean brain levels for expression in both study groups.

miRNA	Group A			Group B		
	Pearson’s *r*	*t*-score	*p*-value	Pearson’s *r*	*t*-score	*p*-value
hsa-let-7d-5p	0.9112	3.1278	0.0444	0.8614	3.3916	0.0137
hsa-miR-30c-5p	0.9065	3.0365	0.0467	0.8982	4.0875	0.0075
hsa-miR-182-5p	0.9877	8.9283	0.0062	0.7669	2.3896	0.0376
hsa-miR-194-5p	0.9041	2.9917	0.0480	0.9743	8.6522	0.0005
hsa-miR-409-5p	0.9960	15.7604	0.0020	0.7943	2.6144	0.0296

To examine wider patterns in the data, we computed pairwise correlations for overall expression of the 362 miRNAs between blood and 14 cortical and subcortical regions in each of the two groups (7 brain regions per group). This was done using Spearman’s rank-order correlations for *mean* tissue-specific miRNA expression levels for the 4 or 6 baboon samples (Table 3). Although all pairwise correlations are highly significant (*P* < 2.2 × 10^–16^), correlations involving PBMC expression are distinctly lower in both groups (*r_*s*_* = 0.42–0.55) than correlations between the brain regions (*r_*s*_* = 0.84–0.99). MiRNA expression in the hypothalamus, paraventricular nuclei (PVN) and cerebellum exhibit the weakest correlations among the pairwise results for the brain regions.

**TABLE 3:** Pairwise Spearman’s correlations (*r*_*s*_ coefficients above diagonal) between blood and brain regions for mean expression counts for study samples per miRNA.

Group A	PBMC	AMG	PVN	dmPFC	dACC	lOFC	mOFC	PCC
PBMC	1	0.48	0.46	0.50	0.42	0.49	0.42	0.49
AMG	0.43,0.53	1	0.95	0.95	0.93	0.96	0.92	0.94
PVN	0.42,0.54	0.93,0.95	1	0.91	0.91	0.92	0.90	0.91
dmPFC	0.46,0.54	0.94,0.96	0.90,0.93	1	0.96	0.99	0.95	0.98
dACC	0.38,0.47	0.91,0.94	0.89,0.92	0.96,0.97	1	0.97	0.99	0.96
lOFC	0.45,0.53	0.95,0.97	0.90,0.93	0.99,0.99	0.96,0.97	1	0.95	0.98
mOFC	0.38,0.46	0.90,0.93	0.88,0.91	0.95,0.96	0.98,0.99	0.95,0.96	1	0.95
PCC	0.45,0.53	0.93,0.95	0.89,0.92	0.98,0.99	0.96,0.97	0.98,0.99	0.95,0.96	1

**Group B**	**PBMC**	**HC**	**CB**	**dlPFC**	**MC**	**EC**	**PPC**	**OC**

PBMC	1	0.55	0.52	0.52	0.54	0.50	0.54	0.55
HC	0.51,0.58	1	0.84	0.96	0.96	0.95	0.97	0.96
CB	0.49,0.56	0.81,0.85	1	0.88	0.87	0.86	0.88	0.85
dlPFC	0.49,0.55	0.95,0.96	0.86,0.89	1	0.98	0.97	0.98	0.94
MC	0.50,0.58	0.96,0.97	0.85,0.89	0.98,0.99	1	0.96	0.99	0.96
EC	0.46,0.53	0.94,0.96	0.84,0.88	0.97,0.98	0.96,0.97	1	0.96	0.95
PPC	0.51,0.58	0.96,0.97	0.86,0.89	0.98,0.98	0.99,0.99	0.96,0.97	1	0.96
OC	0.51,0.58	0.95,0.96	0.83,0.87	0.93,0.95	0.95,0.96	0.94,0.95	0.96,0.97	1

*Expression data were residualized for age for Group A animals and age and sex for Group B animals.*

*Due to the non-normality of the miRNA expression datasets, Spearman’s rank-order method was used for computing pairwise correlations.*

*Estimated r_s_ coefficients are reported above the diagonal; 95% confidence intervals (lower confidence limit, upper confidence limit) are reported below the diagonal.*

*All pairwise correlations presented in the table are highly significant (P < 2.2 × 10^–6^).*

*PBMC, Peripheral blood mononuclear cells; AMG, Amygdala; PVN, Paraventricular nucleus of the hypothalamus; dmPFC, Dorsomedial prefrontal cortex; dACC, Dorsal anterior cingulate cortex; lOFC, Lateral orbitofrontal cortex; mOFC, Medial orbitofrontal cortex; PCC, Posterior cingulate cortex; HC, Hippocampus; CB, Cerebellum; dlPFC, Dorsolateral prefrontal cortex; MC, motor cortex; EC, Entorhinal cortex; PPC, Posterior parietal cortex; OC, Occipital cortex.*

**FIGURE 2:**
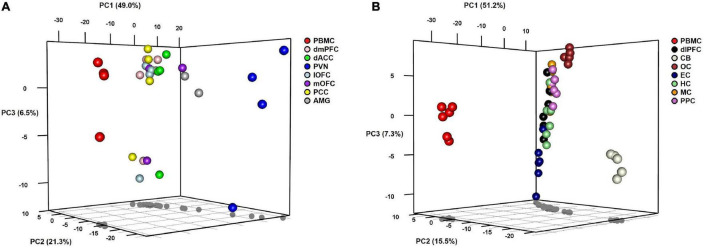
3D plots of results from principal components analyses of miRNA expression levels in baboons by tissue type in **(A)** Group A animals and **(B)** Group B animals.

To visualize variation in miRNA expression between and within samples, we performed principal components analysis (PCA) on samples from each group of baboons (Figure 2). For both groups, the first PC accounts for approximately 50% of the variation between samples and distinguishes PBMCs from the corresponding brain samples along this dimension. In Group A, the PVN appears to be differentiated from the other brain regions for PCs 1 and 2, the latter accounting for ∼21% of the variation. The third PC accounts for ∼6% of the variation, which differentiates the miRNA levels of PBMC and six brain regions of one outlier baboon relative to the other animals in Group A. The AMG, dmPFC, dACC, lOFC, mOFC, and PCC exhibit tight clustering relative to the PBMC and PVN samples in this PC plot. In Group B, PC2 accounts for ∼15% of the variation, and separates the CB, and to a lesser extent the OC, from the other brain regions. The HC, MC, PPC, EC, and dlPFC samples comprise a major cluster in the center of the plot, with notable dispersion along PC3 (explains ∼7% of variation) that exhibits some patterning based on brain region. The independent clustering of the PBMC samples, and to a lesser extent the PVN (Group A) and CB (Group B) samples, is concordant with the pairwise correlations described above. When the normalized miRNA counts of the two groups were lumped together, PCA revealed two tight clusters, one comprised of the PBMC samples from both groups and another of the various brain regions, although the CB samples from Group B again appear as outliers along the third PC (see Supplementary Figure 1).

To quantify the proportion of miRNA variation that is attributable to differences in tissue type, we performed LMM variance components (VC) analysis of the expression data *via* REML (Table 4). For Group A, 71.3% of the variance in miRNA expression is accounted by differences between the 362 miRNAs measured in the dataset. A notable 13.0% of the variance was attributed to tissue type (i.e., blood vs. brain), although it is not significantly different from zero (inferred from the estimated lower 95% confidence limit: VC = 0.14; 95% CI: 0, 0.54). Only 0.6% of the variance was attributed to differences in brain regions (nested within VC for tissue type). For Group B, a similar pattern emerged, with the largest VC estimated for differences between miRNAs (76.7% of total variance). For tissue type and brain region, 7.8% and 0.6% of the variance were attributed to these components, respectively, neither of which are significant. Additionally, VC analysis was performed on the data from both groups, revealing negligible variation attributed to group differences in miRNA expression (see Supplementary Table 2).

**TABLE 4:** Results of variance components analysis of miRNA expression data.

Group A (*n* = 4)			
**Component name**	**Variance component**	**% Total**	95% Confidence interval*
Animal	0.00034	0.03	0, 0.0011
Tissue Type	0.14	13.0	0, 0.54
Tissue Type: Brain Region**	0.0059	0.6	0, 0.013
miRNA	0.77	71.3	0.66, 0.88
Error	0.16	15.1	0.16, 0.17
Total	1.08	100.0	0.76, 1.65

**Group B (*n* = 6)**			
**Component name**	**Variance component**	**% Total**	95% Confidence interval*

Animal	0.000010	0.003	0, 0.00010
Tissue Type	0.083	7.8	0, 0.32
Tissue Type: Brain Region**	0.0066	0.6	0, 0.014
miRNA	0.82	76.7	0.70, 0.94
Error	0.16	14.9	0.16, 0.16
Total	1.07	100.0	0.84, 1.39

*Fixed effects for group A (degrees of freedom based on Satterthwaite’s approximation): intercept = 1.81 (t-value = 5.43; Pr > |t| = 0.029); age = 0.0074 (t-value = 0.52; Pr > |t| = 0.65). For group B: intercept = 2.00 (t-value = 9.16; Pr > |t| = ‘NaN’); age = −0.0026 (t-value = −1.59; Pr > |t| = 0.22); sex = −0.050 (t-value = −2.49; Pr > |t| = 0.12).*

*95% confidence interval: lower confidence limit, upper confidence limit.

**Brain regions nested within tissue type.

**FIGURE 3:**
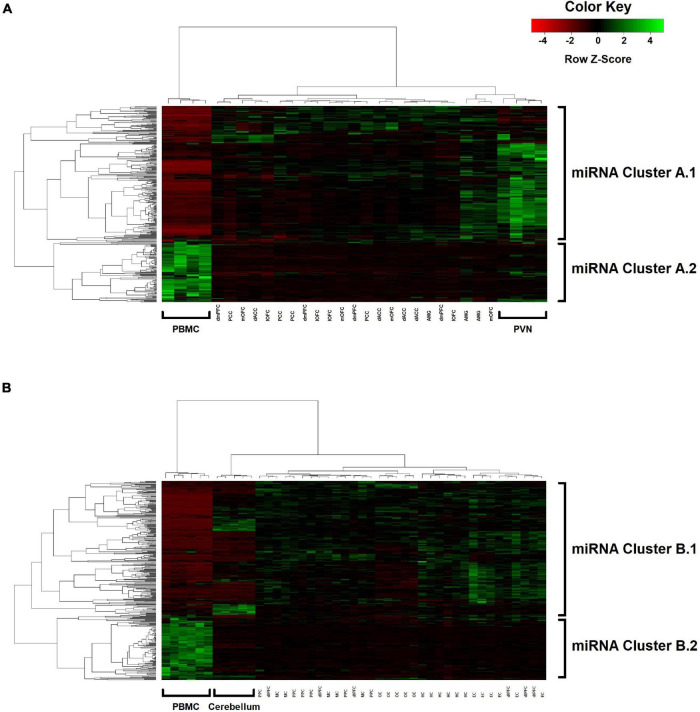
Two-way UPGMA clustering of miRNA expression levels, with aligned heatmap of Z-scores (columns represent the various blood and brain samples; rows represent the 362 miRNAs) in **(A)** Group A animals and **(B)** Group B animals.

Two-way hierarchical clustering was performed to examine the broader co-expression patterns among the 362 miRNAs and how these patterns may differ between blood and the various brain regions (Figure 3). For Group A (Figure 3A), the dendrogram shown at the top represents a UPGMA cluster of the 31 blood and brain samples based on correlation distances determined by Spearman’s rank-order method, with the four PBMC samples positioned together as an outlier branch. The dendrogram shown on the left margin represents a UPGMA cluster of the 362 miRNAs based on Pearson correlation distances, with what appears to be two major branches, designated here as A.1 (*n* = 250 miRNAs; median *r* = 0.29 between blood and mean brain expression levels) and A.2 (*n* = 112 miRNAs; median *r* = 0.19). The tissue samples and miRNAs represented by the nodes in the two dendrograms are aligned as columns and rows in the heatmap, respectively, and intersect within a color-coded matrix of Z-scores for miRNA expression levels across the samples. Interestingly, branches A.1 and A.2 in the miRNA dendrogram reveal distinctly different expression patterns among the PBMC samples, with A.1 broadly showing elevated expression relative to the brain, whereas miRNAs in the other branch exhibit depressed levels. Furthermore, most of the miRNAs within the A.1 branch show lower levels of expression in the hypothalamus relative to the other brain regions.

To identify potential biological signatures in the two miRNA branches, MSEA was performed using the web-based miEAA application, with miRNA lists ordered according to Pearson correlations between PBMC and mean brain expression levels (i.e., we are not examining miRNA expression levels *per se*, but how they correlate between blood and brain samples and whether highly correlated miRNAs are enriched in particular biological pathways and ontologies). The top enriched miRNA category for branch A.1 is “CD14 expressed” (FDR-adjusted *P* = 8.7 × 10^–4^; *n* = 121 miRNAs, listed in Supplementary Table 3), with three other immunity-related categories among the four next most significant enrichment results (FDR-adjusted *P* < 0.01; see Table 5). For A.2, no significant enrichments were observed.

**TABLE 5:** Top-5 MSEA results for miRNAs representing dendrogram branches A.1 and B.1.

Dendrogram branch A.1 (*n* = 250 miRNAs)			
miEAA category*	**Subcategory**	**FDR-adjusted *P*-value^Ψ^**	**No. observed**
Immune cells	CD14 expressed	8.7 × 10^–4^	121^§^
Immune cells	CD15 expressed	1.9 × 10^–3^	138
Gene ontology**	Lymphocyte proliferation	3.5 × 10^–3^	15
Immune cells	CD3 expressed	3.8 × 10^–3^	105
Immune cells	CD56 expressed	4.9 × 10^–3^	121

**Dendrogram branch B.1 (*n* = 243 miRNAs)**			
**miEAA category**	**Subcategory**	**FDR-adjusted *P*-value**	**No. observed**

Immune cells	CD14 expressed	2.0 × 10^–3^	121^§^
Immune cells	CD15 expressed	2.3 × 10^–3^	137
Immune cells	CD19 expressed	2.3 × 10^–3^	103
Immune cells	CD3 expressed	2.3 × 10^–3^	105
Immune cells	CD56 expressed	2.3 × 10^–3^	126

*MiRNA Set Enrichment Analysis (MSEA) was performed on lists of miRNA sorted by Pearson correlation coefficients estimated between PBMC expression levels and mean expression levels across seven brain regions (within each study group).*

*Immune cell data from Leidinger et al. (2014) and comprises miRNAs expressed in at least three individuals for respective cell types (CD14+, CD15+, CD19+, CD3+, and CD56+).

**Data from miRWalk 2.0 (Dweep et al., 2011).

*^Ψ^Adjustment using Benjamini–Hochberg approach.*

*^§^Observed miRNAs for “CD14 expressed” listed in Supplementary Table 3.*

For Group B (Figure 3B), similar patterns emerge in the two-way hierarchical clusters and heatmap. The PBMC samples are an outlier branch in the top dendrogram, as well as samples from the cerebellum, which is consistent with the PCA results. Two major branches are again evident in the miRNA dendrogram (left margin): upregulated B.1 (*n* = 243 miRNAs; median *r* = 0.19 between blood and mean brain expression levels) and downregulated B.2 (*n* = 119 miRNAs; median *r* = 0.12). MSEA for B.1 (Table 5) shows a similar enrichment pattern to A.1, with the top enriched category again being “CD14 expressed” (FDR-adjusted *P* = 2.0 × 10^–3^; *n* = 121 miRNAs), with four other immunity-related categories rounding out the top-five. For B.2, two significant enrichments were observed, both related to Alzheimer’s Disease (AD): “Alzheimer’s Disease deregulated” (FDR-adjusted *P* = 2.7 × 10^–3^; *n* = 25 miRNAs) and “Alzheimer’s Disease upregulated” (FDR-adjusted *P* = 0.013; *n* = 12 miRNAs). Comparing the miRNA dendrograms from Groups A and B, highly significant overlap was observed for upregulated branches A.1 and B.1 (*n* = 187 shared miRNAs out of 250 and 243 miRNAs, respectively; Fisher’s exact test *P* = 4.6 × 10^–6^), with only approximately half of the miRNAs shared between A.2 and B.2.

Given the well-established regulatory role played by miRNAs in gene expression, the biological implications of the matching A.1 and B.1 clusters were further explored by examining the gene targets of the constituent miRNAs (Supplementary Table 4). For both clusters, the top-enriched gene target is *MYC* (A.1: target of 46 miRNAs, FDR-adjusted *P* = 7.3 × 10^–14^; B.1: target of 45 miRNAs, FDR-adjusted *P* = 7.3 × 10^–14^). Also, among the top-five gene targets are *AGO1* and *EEF1A1* for both clusters, underscoring their overlap in miRNAs. For lists of enriched gene targets with FDR-adjusted *P* < 0.01 (*n* = 1,489 and 1,058, respectively), functional assessment based on the Gene Ontology (GO) database showed the most significant annotations to be the same for both clusters: protein binding (A.1: 1,004 genes, FDR-adjusted *P* = 7.3 × 10^–57^; B.1: 730 genes, FDR-adjusted *P* = 1.6 × 10^–43^); poly(A) RNA binding (A.1: 212 genes, FDR-adjusted *P* = 2.7 × 10^–29^; B.1: 153 genes, FDR-adjusted *P* = 2.0 × 10^–20^); and positive regulation of transcription from RNA polymerase II promoter (A.1: 149 genes, FDR-adjusted *P* = 6.1 × 10^–10^; B.1: 127 genes, FDR-adjusted *P* = 6.0 × 10^–14^).

## Discussion

The relationship between blood and brain miRNA expression activity is not well understood. With various peripheral miRNAs implicated as potential biomarkers for neurological and psychiatric pathologies, it is necessary to discern how these correlate with miRNA expression levels in the brain that are presumably more proximate to underlying etiological mechanisms. In the present study we computed cross-tissue correlations and used PCA, variance components analysis, hierarchical clustering, and enrichment analysis to better understand the relationship between miRNA expression in peripheral blood and 14 cortical (dmPFC, dlPFC, dACC, lOFC, mOFC, PCC, MC, EC, PPC, OC) and subcortical (AMG, PVN, HC, CB) brain regions in baboons. To our knowledge, this is the first study to investigate the transcriptome-wide relationship between blood and brain miRNA expression using *paired* (i.e., within-subject) samples.

Although significantly correlated, our analyses show that miRNA expression profiles from PBMCs are differentiated from corresponding profiles in the brain, with certain brain regions, in particular the cerebellum, exhibiting divergent expression patterns. From Spearman’s pairwise correlation analysis, we found moderate, although highly significant, tissue-wide correlations between miRNAs expressed in blood and in the brain (*r_*s*_* = 0.42–0.55). Prior studies have examined *gene expression* profiles of *independent* blood and brain samples (i.e., from different human subjects), with one study reporting correlations between leukocytes and three brain regions that are lower than our pairwise results (*r* = 0.29–0.32) (Cai et al., 2010); whereas another reported more similar levels of correlation for whole blood and 16 brain regions (Spearman’s *r_*s*_* = 0.51; range *r_*s*_* = 0.44–0.58) (Sullivan et al., 2006). For within-subject correlations of human gene expression the data is more limited, although Rollins et al. (2010) computed a Pearson’s correlation of *r* = 0.64 for PBMCs and cerebellar tissue (Rollins et al., 2010). And in a non-human primate study with a paired sample design similar to the one used here, Jasinska et al. (2009) reported that 429 of 2,481 transcript probes expressed in 12 male vervet monkeys showed blood-brain correlations of *r_*s*_* > 0.55 for at least one of eight examined brain regions (Jasinska et al., 2009). Overall, these results suggest that the levels of correlation between peripheral blood and the brain are broadly similar for miRNA and gene expression activity, likely reflecting the close regulatory relationship between these molecules.

In the tissue-based dendrograms and PCA plots, however, the PBMC samples from both Group A and B animals appear as outliers, with variance components analysis attributing about 10% of the variation in miRNA expression levels to differences between PBMCs and the brain regions (with most stemming from differences between individual miRNAs). Notably, the corresponding miRNA-based dendrograms for each study group appear to mirror each other, both harboring major branches (A.1 and B.1) with extensive upregulation among PBMCs and enrichment for immune-expressed miRNAs among the strongest blood-brain correlates. Given the tree-building UPGMA algorithm is based on a correlation distance, the tightly clustered sub-branches that comprise A.1 and B.1 are indicative of strong co-expression (i.e., linear relationships) between individual miRNA profiles across both the blood and brain samples, although the PBMC expression levels, as highlighted by the heatmap Z-scores, are distinctly elevated relative to the brain tissues (and vice versa for the A.2 and B.2 branches).

MSEA analysis of the A.1 and B.1 branches show an enrichment of miRNAs expressed in CD14+, CD15+, CD19+, CD3+, and CD56+ immune cell types, which are known markers of neuroinflammation and have been increasingly implicated in neurodegenerative and psychiatric disorders. The most significant enrichment in both study groups are for miRNAs expressed in immune cells harboring CD14, a receptor shown to mediate neuroinflammation induced by Alzheimer’s amyloid β peptides (Fassbender et al., 2004) and cerebral ischemia in a mouse model (Zhou et al., 2013). Increased CD14 + levels in serum and cerebrospinal fluid (CSF) have also been associated with schizophrenia and bipolar disorder risk (Jakobsson et al., 2015; Johansson et al., 2017). Similarly, for the other immune cell types implicated in the MSEA analysis of miRNA blood-brain correlates, significant aberrations in cell subpopulation levels have been reported for various neurological and psychiatric conditions, both in brain tissue (Busse et al., 2012; Geng and Huang, 2021; Troscher et al., 2021) and peripheral blood samples (Schleifer et al., 2002; Richartz-Salzburger et al., 2007; Miller et al., 2013; Cacabelos et al., 2016; Rocha et al., 2018; Furlan et al., 2019; Pietruczuk et al., 2019), including major depression, schizophrenia, epilepsy, bipolar disorder and Parkinson’s disease, among others, further suggesting a broad, pathophysiological role for changes in inflammation profiles.

The gene targets of the miRNAs comprising the A.1 and B.1 dendrogram branches also provide insight. For both, *MYC* is the top enriched gene, the target of 46 and 45 miRNAs, respectively. The gene encodes c-Myc, a nuclear phosphoprotein and “master” transcription factor that regulates growth-related genes, including *VEGFA* and biological pathways that promote angiogenesis and endothelial cell growth (Shi et al., 2014; Kim et al., 2015). Interestingly, in a recent study examining neural stem cells (NSC) and their transition from quiescence to activation, c-Myc was found to be dynamically expressed during the transition, with increased levels during activation that coordinate cell cycle and mitochondrial reprogramming (Cai et al., 2021). NSC activation switching is a focal regulatory point for neurogenesis and promotes plasticity in existing circuitry by making new synaptic contacts with mature neurons (Bond et al., 2015; Urban et al., 2019), with implications for learning, memory formation, and executive function. GO-term annotation analysis of *MYC* and the other top gene targets for the A.1 and B.1 miRNAs showed enrichment for gene functions related to protein and poly(A) binding and transcription regulation, a likely signature of the complex, multilevel regulation of gene expression by miRNAs (Makeyev and Maniatis, 2008).

Transcriptomic profiles have been reported for different regions within the brain, revealing distinct gene expression patterns, suggesting that any blood-brain relationship in miRNA expression is likely varied by brain tissue type. Analyzing microarray data for six neurotypical adults and 191 brain structures from the Allen Brain Atlas, Negi and Guda (2017) demonstrated through hierarchical clustering that the cerebellum has the most distinguishable gene expression patterns compared to other brain regions (Negi and Guda, 2017), with tissue-specific blocks of up- and downregulated genes. This is consistent with the hierarchical clustering of miRNA expression presented here, as the cerebellum also exhibits distinctive signatures of up- and downregulated miRNAs, and whose outlier status among brain regions is further evident from the PCA plots. Within the major A.1 and B.1 dendrogram clusters, the cerebellum samples exhibit strong upregulation for three particular subclusters (*n* = 109 miRNAs), corresponding to the wider upregulation observed for the PBMC samples. Gene targets were re-identified for the miRNAs comprising these subclusters, with *MYC* again emerging as the top-enriched target (FDR-adjusted *P* = 9.9 × 10^–5^). GO-term annotation analysis of the top targets (*n* = 116; FDR-adjusted *P* < 0.01) showed the most significant functional enrichment to be again for protein binding (FDR-adjusted *P* = 2.5 × 10^–6^), as well as positive regulation of smooth muscle cell proliferation (FDR-adjusted *P* = 1.5 × 10^–4^), a key component of regulating neurovascular contractility and blood-brain barrier integrity (Hill et al., 2015).

Lastly, we examined expression levels of individual miRNAs for correlations between blood and brain (using the mean of the different regions) to better understand their suitability as peripheral biomarkers for brain-related conditions. However, given the small sample sizes and hence limited statistical power, only one miRNA (hsa-miR-99b-5p) emerged as significantly correlated (after Bonferroni correction) between blood and brain samples from Group A animals. In humans, increased expression of hsa-miR-99b-5p within PBMCs has been associated with pediatric multiple sclerosis (Liguori et al., 2018). miR-99b-5p has also been found to be abnormally expressed in the brains of a mouse model for Alzheimer’s disease, affecting neuron survival *via* the mTOR pathway in response to amyloid β-induced endoplasmic reticulum stress (Ye et al., 2015) and has been implicated in rat models of stress resilience (Chen et al., 2015) and transient cerebral ischemia (Altintas et al., 2016). We identified a limited number of miRNAs showing *nominally* significant blood-brain correlations across both study groups (hsa-let-7d-5p, hsa-miR-30c-5p, hsa-miR-182-5p, hsa-miR-194-5p and hsa-miR409-5p), all of which have been previously implicated as peripheral biomarkers of various psychiatric or neurological disorder, or within animal models of brain-related disorders (e.g., Zhou et al., 2009; Tan et al., 2014; Vallelunga et al., 2014; Wang et al., 2015; Maffioletti et al., 2016; Toyama et al., 2017; Bahi and Dreyer, 2020; Brennan et al., 2020; Qi et al., 2020; Levitskiy et al., 2021). However, the permissive statistical significance level of these miRNA correlations precludes any firm conclusions to be drawn about their suitability as potential peripheral biomarkers.

Overall, the results of the study reveal significant blood-brain correlations in miRNA expression profiles in baboons on a tissue-wide level, but with evidence of some differentiation, as the pairwise correlations among the 14 examined brain regions are markedly stronger, indicated by the outlying positions of PBMC samples within UPGMA dendrograms and PCA plots. However, a major miRNA co-expression cluster was identified in the dendrograms of each study group, both exhibiting global upregulation among PBMCs and enrichment for miRNAs expressed in immune cell types, with gene targets functionally annotated for different aspects of gene expression regulation. The study is limited by its small sample size and the assessment of only peripheral blood cells, as other sources (e.g., plasma, CSF, saliva, urine) may provide additional insight into the use of peripheral tissues as biomarkers of brain-related miRNA expression. Thus, future studies investigating this research question should aim to profile and analyze miRNA expression in a wider array of peripheral tissues from a larger cohort of animals. Nonetheless, the data generated here provides preliminary evidence of blood-brain correlations and that changes in peripheral miRNA expression patterns, including ones that potentially impact neuroinflammation, multilevel gene regulation, and c-Myc-mediated processes, may also reflect changes in the brain within the same subject.

## Data Availability Statement

The datasets presented in this study can be found in online repositories. The names of the repository/repositories and accession number(s) can be found below: https://www.ncbi.nlm.nih.gov/search/all/?term=GSE190675.

## Ethics Statement

The animal study was reviewed and approved by Texas Biomedical Research Institute Institutional Animal Care and Use Committee (IACUC).

## Author Contributions

MC devised the project and coordinated RNA extraction and miRNA sequencing. DC and CL performed the tissue collection and brain dissection. MK, SP, and JN processed the miRNA expression data and performed statistical analyses. MK and MC took the lead in writing the manuscript with assistance from SP, AK, and ED. DC, AK, CL, and PN provided critical feedback that helped shape the study design and the conclusions drawn from the data. All authors contributed to the article and approved the submitted version.

## Conflict of Interest

The authors declare that the research was conducted in the absence of any commercial or financial relationships that could be construed as a potential conflict of interest.

## Publisher’s Note

All claims expressed in this article are solely those of the authors and do not necessarily represent those of their affiliated organizations, or those of the publisher, the editors and the reviewers. Any product that may be evaluated in this article, or claim that may be made by its manufacturer, is not guaranteed or endorsed by the publisher.
