# Supplementary figures and images for: Blood-Based miRNA Biomarkers as Correlates of Brain-Based miRNA Expression
Source: Front Mol Neurosci. 2022 Mar 22;15:817290. doi: 10.3389/fnmol.2022.817290 (PMC8981579; doi:10.3389/fnmol.2022.817290)

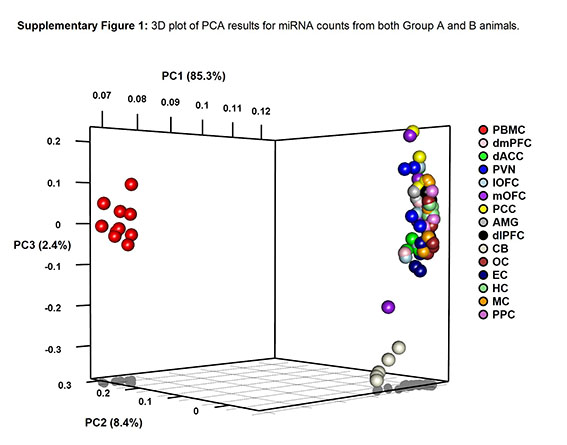

Supplement: Supplementary file 1 [file Image_1.JPEG]

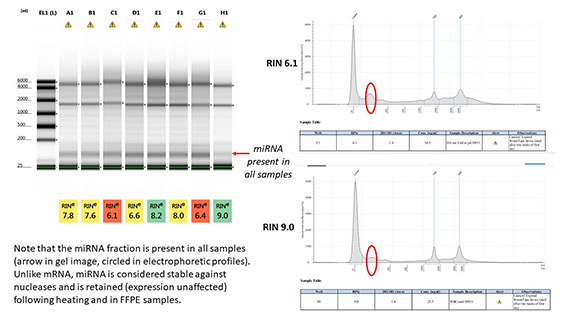

Supplement: Supplementary file 2 [file Image_2.JPEG]
