# Supplementary material for: Blood-Based miRNA Biomarkers as Correlates of Brain-Based miRNA Expression
Source: Front Mol Neurosci. 2022 Mar 22;15:817290. doi: 10.3389/fnmol.2022.817290 (PMC8981579; doi:10.3389/fnmol.2022.817290)
Supplement: Supplementary file 4 [file Table_2.DOCX]

**Supplementary Table 2.** Results of variance components analysis of miRNA expression data for Groups A and B combined.

| **Group A and B (n=10)** | | | |
| --- | --- | --- | --- |
| **Component Name** | **Variance Component** | **% Total** | **95% Confidence Interval*** |
| Group | 0 | 0 | 0, 0 |
| Group: Animal** | 0.00010 | 0.009 | 0, 0.00030 |
| Tissue Type | 0.11 | 10.2 | 0, 0.42 |
| Tissue Type: Brain Region** | 0.0090 | 0.8 | 0.0020, 0.016 |
| miRNA | 0.79 | 73.8 | 0.67, 0.90 |
| Error | 0.17 | 15.9 | 0.17, 0.17 |
| TOTAL | 1.07 | 100.0 | 0.81, 1.50 |

Fixed effects: intercept=2.05; age=-0.0031; sex=-0.061.

* 95% confidence interval: lower confidence limit, upper confidence limit.

** Animal subjects nested within study group, and brain regions nested within tissue type.
