# Supplementary material for: Blood-Based miRNA Biomarkers as Correlates of Brain-Based miRNA Expression
Source: Front Mol Neurosci. 2022 Mar 22;15:817290. doi: 10.3389/fnmol.2022.817290 (PMC8981579; doi:10.3389/fnmol.2022.817290)
Supplement: Supplementary file 6 [file Table_4.DOCX]

**Supplementary Table 4.** Top-five enriched gene targets among miRNAs comprising UPGMA dendogram clusters A.1 and B.1.

| **Cluster A.1** | | | | |
| --- | --- | --- | --- | --- |
| **Gene Target** | **Observed*** | **Expected** | ***P*-value**** | **miRNAs** |
| *MYC* | 46 | 11.8 | 7.3 × 10^-14^ | hsa-let-7i-5p; hsa-miR-451a; hsa-miR-423-5p; hsa-miR-16-2-3p; hsa-miR-92a-3p; hsa-miR-378a-3p; hsa-miR-148a-3p; hsa-miR-148a-5p; hsa-miR-93-5p; hsa-let-7f-5p; hsa-miR-25-3p; hsa-miR-30d-3p; hsa-miR-20a-5p; hsa-miR-17-5p; hsa-miR-23a-3p; hsa-miR-126-5p; hsa-miR-186-5p; hsa-miR-155-5p; hsa-miR-185-5p; hsa-miR-30e-3p; hsa-miR-19a-3p; hsa-miR-21-5p; hsa-miR-106b-5p; hsa-miR-16-5p; hsa-let-7g-5p; hsa-miR-24-3p; hsa-let-7a-5p; hsa-let-7d-5p; hsa-miR-29a-3p; hsa-miR-129-2-3p; hsa-miR-335-5p; hsa-miR-487b-3p; hsa-miR-323a-3p; hsa-miR-130a-3p; hsa-miR-377-5p; hsa-miR-212-3p; hsa-miR-125a-3p; hsa-let-7e-5p; hsa-miR-34a-5p; hsa-miR-33a-5p; hsa-miR-30c-5p; hsa-miR-30d-5p; hsa-miR-33b-5p; hsa-let-7b-5p; hsa-let-7c-5p; hsa-miR-324-3p |
| *AGO1* | 43 | 11.1 | 7.3 × 10^-13^ | hsa-let-7i-5p; hsa-miR-423-3p; hsa-miR-92a-3p; hsa-miR-93-3p; hsa-miR-378a-3p; hsa-miR-484; hsa-miR-19b-3p; hsa-miR-93-5p; hsa-let-7f-5p; hsa-miR-25-3p; hsa-miR-194-5p; hsa-miR-652-3p; hsa-miR-20a-5p; hsa-miR-17-5p; hsa-miR-186-5p; hsa-miR-148b-3p; hsa-miR-185-5p; hsa-miR-130b-3p; hsa-miR-19a-3p; hsa-miR-106b-5p; hsa-let-7g-5p; hsa-let-7a-5p; hsa-let-7d-5p; hsa-miR-29a-3p; hsa-miR-326; hsa-miR-197-3p; hsa-miR-193b-3p; hsa-miR-628-3p; hsa-miR-324-5p; hsa-miR-107; hsa-miR-877-5p; hsa-miR-149-5p; hsa-miR-421; hsa-miR-1251-5p; hsa-miR-212-3p; hsa-let-7e-5p; hsa-miR-34a-5p; hsa-miR-30c-5p; hsa-miR-30d-5p; hsa-let-7b-5p; hsa-let-7c-5p; hsa-miR-328-3p; hsa-miR-330-3p |
| *HSPA1B* | 41 | 11.0 | 1.4 × 10^-11^ | hsa-miR-7977; hsa-miR-130b-5p; hsa-miR-423-3p; hsa-miR-92a-3p; hsa-miR-378a-3p; hsa-miR-425-3p; hsa-miR-10a-5p; hsa-miR-484; hsa-miR-25-3p; hsa-miR-7-1-3p; hsa-miR-652-3p; hsa-miR-340-5p; hsa-miR-424-5p; hsa-miR-15b-5p; hsa-miR-505-3p; hsa-miR-339-5p; hsa-miR-15a-5p; hsa-miR-142-3p; hsa-miR-106b-5p; hsa-miR-16-5p; hsa-miR-331-3p; hsa-miR-326; hsa-miR-664a-3p; hsa-miR-197-3p; hsa-miR-193b-3p; hsa-miR-324-5p; hsa-miR-149-5p; hsa-miR-335-5p; hsa-miR-361-5p; hsa-miR-495-3p; hsa-miR-212-3p; hsa-miR-330-5p; hsa-miR-181c-5p; hsa-miR-181d-5p; hsa-miR-34a-5p; hsa-miR-1296-5p; hsa-miR-30d-5p; hsa-miR-22-3p; hsa-let-7b-5p; hsa-miR-328-3p; hsa-miR-324-3p |
| *DNMT1* | 23 | 3.7 | 2.7 × 10^-11^ | hsa-miR-1260b; hsa-miR-92a-3p; hsa-miR-148a-3p; hsa-miR-484; hsa-miR-19b-3p; hsa-miR-342-3p; hsa-miR-20a-5p; hsa-miR-17-5p; hsa-miR-126-3p; hsa-miR-18a-5p; hsa-miR-155-5p; hsa-miR-148b-3p; hsa-miR-185-5p; hsa-miR-140-5p; hsa-miR-19a-3p; hsa-miR-29a-3p; hsa-miR-30b-5p; hsa-miR-193b-3p; hsa-miR-149-5p; hsa-miR-377-3p; hsa-miR-30c-5p; hsa-miR-152-3p; hsa-let-7c-5p |
| *EEF1A1* | 29 | 6.5 | 4.4 × 10^-10^ | hsa-miR-423-3p; hsa-miR-1260b; hsa-miR-92a-3p; hsa-miR-93-3p; hsa-miR-484; hsa-miR-93-5p; hsa-miR-25-3p; hsa-miR-652-3p; hsa-miR-15b-5p; hsa-miR-17-5p; hsa-miR-18a-5p; hsa-miR-186-5p; hsa-miR-26b-5p; hsa-miR-185-5p; hsa-miR-505-3p; hsa-miR-339-5p; hsa-miR-30e-5p; hsa-miR-106b-5p; hsa-miR-16-5p; hsa-miR-361-3p; hsa-let-7g-5p; hsa-miR-340-3p; hsa-miR-24-3p; hsa-miR-197-3p; hsa-miR-877-5p; hsa-miR-421; hsa-miR-30c-5p; hsa-let-7b-5p; hsa-miR-324-3p |
| **Cluster B.1** | | | | |
| **Gene Target** | **Observed*** | **Expected** | ***P*-value**** | **miRNAs** |
| *MYC* | 45 | 11.3 | 8.0 × 10^-14^ | hsa-let-7i-5p; hsa-let-7d-5p; hsa-let-7f-5p; hsa-miR-126-5p; hsa-let-7g-5p; hsa-miR-148a-3p; hsa-miR-148a-5p; hsa-miR-30e-3p; hsa-miR-186-5p; hsa-miR-19a-3p; hsa-miR-16-5p; hsa-miR-25-3p; hsa-miR-21-5p; hsa-miR-378a-3p; hsa-miR-185-5p; hsa-miR-92a-3p; hsa-miR-93-5p; hsa-miR-106b-5p; hsa-miR-17-5p; hsa-miR-20a-5p; hsa-miR-423-5p; hsa-miR-23a-3p; hsa-miR-155-5p; hsa-miR-16-2-3p; hsa-miR-30d-3p; hsa-miR-24-3p; hsa-miR-29a-3p; hsa-let-7a-5p; hsa-miR-33a-5p; hsa-miR-320b; hsa-miR-455-3p; hsa-miR-7-5p; hsa-miR-30c-5p; hsa-miR-30d-5p; hsa-miR-222-3p; hsa-miR-135a-5p; hsa-miR-130a-3p; hsa-miR-323a-3p; hsa-miR-487b-3p; hsa-miR-377-5p; hsa-miR-324-3p; hsa-miR-34c-5p; hsa-miR-451a; hsa-miR-33b-5p; hsa-miR-151a-5p |
| *AGO1* | 39 | 10.6 | 2.2 × 10^-10^ | hsa-let-7i-5p; hsa-let-7d-5p; hsa-miR-484; hsa-let-7f-5p; hsa-let-7g-5p; hsa-miR-148b-3p; hsa-miR-186-5p; hsa-miR-19a-3p; hsa-miR-19b-3p; hsa-miR-25-3p; hsa-miR-378a-3p; hsa-miR-185-5p; hsa-miR-130b-3p; hsa-miR-197-3p; hsa-miR-92a-3p; hsa-miR-93-5p; hsa-miR-106b-5p; hsa-miR-652-3p; hsa-miR-17-5p; hsa-miR-20a-5p; hsa-miR-423-3p; hsa-miR-93-3p; hsa-miR-29a-3p; hsa-miR-26a-5p; hsa-let-7a-5p; hsa-miR-194-5p; hsa-miR-455-3p; hsa-miR-30c-5p; hsa-miR-30d-5p; hsa-miR-146b-5p; hsa-miR-421; hsa-miR-628-3p; hsa-miR-1251-5p; hsa-miR-103a-3p; hsa-miR-218-5p; hsa-miR-219a-2-3p; hsa-miR-326; hsa-miR-151a-5p; hsa-miR-138-5p |
| *PTEN* | 40 | 11.3 | 2.5 × 10^-10^ | hsa-miR-182-5p; hsa-miR-486-5p; hsa-miR-10a-5p; hsa-miR-19a-3p; hsa-miR-19b-3p; hsa-miR-32-5p; hsa-miR-142-5p; hsa-miR-25-3p; hsa-miR-21-3p; hsa-miR-21-5p; hsa-miR-130b-3p; hsa-miR-18a-5p; hsa-miR-92a-3p; hsa-miR-93-5p; hsa-miR-425-5p; hsa-miR-106b-3p; hsa-miR-106b-5p; hsa-miR-17-3p; hsa-miR-17-5p; hsa-miR-20a-5p; hsa-miR-23a-3p; hsa-miR-155-5p; hsa-miR-26b-5p; hsa-miR-29a-3p; hsa-miR-22-3p; hsa-miR-26a-5p; hsa-miR-29c-3p; hsa-miR-29a-5p; hsa-miR-105-5p; hsa-miR-222-3p; hsa-miR-221-3p; hsa-miR-103a-3p; hsa-miR-377-3p; hsa-miR-130a-3p; hsa-miR-543; hsa-miR-382-5p; hsa-miR-376a-5p; hsa-miR-181a-5p; hsa-miR-181b-5p; hsa-miR-144-3p |
| *EEF1A1* | 29 | 6.3 | 2.6 × 10^-10^ | hsa-miR-484; hsa-miR-340-3p; hsa-let-7g-5p; hsa-miR-30e-5p; hsa-miR-186-5p; hsa-miR-16-5p; hsa-miR-25-3p; hsa-miR-361-3p; hsa-miR-185-5p; hsa-miR-339-5p; hsa-miR-197-3p; hsa-miR-1260b; hsa-miR-18a-5p; hsa-miR-92a-3p; hsa-miR-93-5p; hsa-miR-106b-5p; hsa-miR-652-3p; hsa-miR-15b-5p; hsa-miR-17-5p; hsa-miR-423-3p; hsa-miR-26b-5p; hsa-miR-505-3p; hsa-miR-93-3p; hsa-miR-24-3p; hsa-miR-455-3p; hsa-miR-30c-5p; hsa-miR-421; hsa-miR-221-3p; hsa-miR-324-3p |
| *BCL2* | 28 | 6.3 | 1.4 × 10^-9^ | hsa-miR-182-5p; hsa-miR-126-3p; hsa-miR-148a-3p; hsa-miR-15a-5p; hsa-miR-16-5p; hsa-miR-21-5p; hsa-miR-18a-5p; hsa-miR-15b-5p; hsa-miR-17-5p; hsa-miR-20a-5p; hsa-miR-24-2-5p; hsa-miR-29a-3p; hsa-miR-29c-3p; hsa-let-7a-5p; hsa-miR-192-5p; hsa-miR-204-5p; hsa-miR-30b-5p; hsa-miR-7-5p; hsa-miR-139-5p; hsa-miR-135a-5p; hsa-miR-103a-3p; hsa-miR-376c-3p; hsa-miR-34c-5p; hsa-miR-211-5p; hsa-miR-181a-5p; hsa-miR-181b-5p; hsa-miR-451a; hsa-miR-33b-5p |

Gene targets of miRNAs comprising UPGMA dendrogram clusters A.1 and B.1 were identified from miRTarBase, a curated database of miRNA-target interactions. Over-representation analysis (i.e., Fisher’s exact test) was performed on these target lists using the online tool miEAA.

*Number of miRNAs in A.1 or B.1 cluster that target gene.

**Adjustment using Benjamini-Hochberg approach.
